# Idiopathic pigmented vitreous cyst without autofluorescence: a case report

**DOI:** 10.1186/s12886-017-0580-6

**Published:** 2017-10-03

**Authors:** Jing Lu, Yan Luo, Lin Lu

**Affiliations:** State Key Laboratory of Ophthalmology, Zhongshan Image Reading Center, Zhongshan Ophthalmic Center, Sun Yat-Sen University, Guangzhou, 510060 China

**Keywords:** Vitreous cyst, Autofluorescence, Anterior segment optical coherence tomography (AS-OCT), Case report

## Abstract

**Background:**

Vitreous cysts are rare clinical findings and seldom cause visual disturbance. They are generally classified as congenital or acquired and are considered idiopathic when the etiology can not be determined. A previous electron microscopic observation on an idiopathic pigmented vitreous cyst has confirmed its pigment epithelial origin. However, the specific kind of pigment epithelium involved remains unclear.

**Case presentation:**

A 39-year-old female presented with a round-shaped floater causing frequent visual disturbance in the left eye. A pigmented, non lobulated and freely mobile vitreous cyst was observed in the anterior vitreous by slit lamp examination and anterior segment optical coherence tomography. The pigment clumps on the cyst wall showed no autofluorescence. No persistent hyaloid artery or connection between the cyst and ocular structures was found by fundus fluorescein angiography and B-scan ultrasound. Serum tests for cysticercoids, sparganosis and toxoplasma were negative. A diagnosis of idiopathic vitreous cyst was made and no intervention was given. The cyst sank to the inferior part of the vitreous and the patient felt less visual disturbance during one-year follow-up.

**Conclusions:**

We described the features of a pigmented vitreous cyst revealed by autofluorescence and anterior segment optical coherence tomography for the first time. The intact retina, the absence of lipofuscin of the cyst and its location in the anterior vitreous led to the hypothesis that the cyst may originate from the ciliary pigment epithelium rather than the retinal pigment epithelium.

## Background

Vitreous cysts are rare ocular malformations, which can be pigmented or non-pigmented; free floating or fixed; spherical, oval or lobulated [1]. The mean age of patients with vitreous cysts ranges from 5 to 68 years [1]. Most of the vitreous cysts are asymptomatic and rarely require intervention with sizes from 0.15 mm to 12 mm. When they become visually disturbing, argon [2, 3] or Nd-YAG [4, 5] laser photocystotomy and pars plana vitrectomy [6–8] can be applied. Here, we described the features of an idiopathic pigmented vitreous cyst by autofluorescence and anterior segment optical coherence tomography (AS-OCT) for the first time.

## Case presentation

A 39-year-old Chinese female noted a round-shaped floater in the left eye 7 years ago and had experienced frequent visual disturbances for 3 months. Her medical history was unremarkable except for a surgery of laser in situ keratomileusis on both eyes 17 years ago. Her best corrected visual acuity was 20/20 OU. Axial length was 24.4 mm OD and 24.8 mm OS. A free-floating pigmented vitreous cyst was observed in the anterior vitreous of the left eye under the slit lamp. Except for the cyst, her bilateral ocular examination was unremarkable. No degeneration of the peripheral retina was detected in both eyes.

**Fig. 1:**
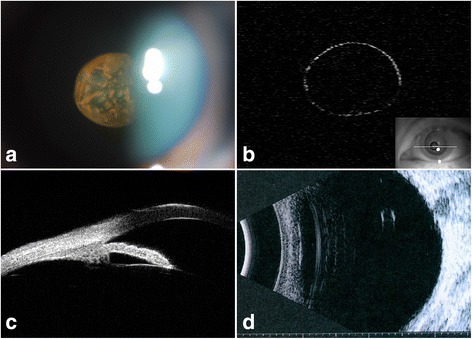
**a** Anterior segment photograph showing a spherical translucent vitreous cyst with irregular yellowish-brown pigmentation on its surface. **b** Anterior segment-optical coherence tomography showing a nonlobulated cyst, which had a thin cyst wall with punctuated thickening. **c** Ultrasound biomicroscopy showing ciliary body and posterior iris without obvious anomalies. **d** The cyst shifted to the posterior vitreous when the patient was in a supine position undergoing B-scan ultrasound, which showed a 2.2 mm × 1.5 mm hyperechogenic spherical structure without calcification, internal reflectivity, or attachment to ocular structures

**Fig. 2:**
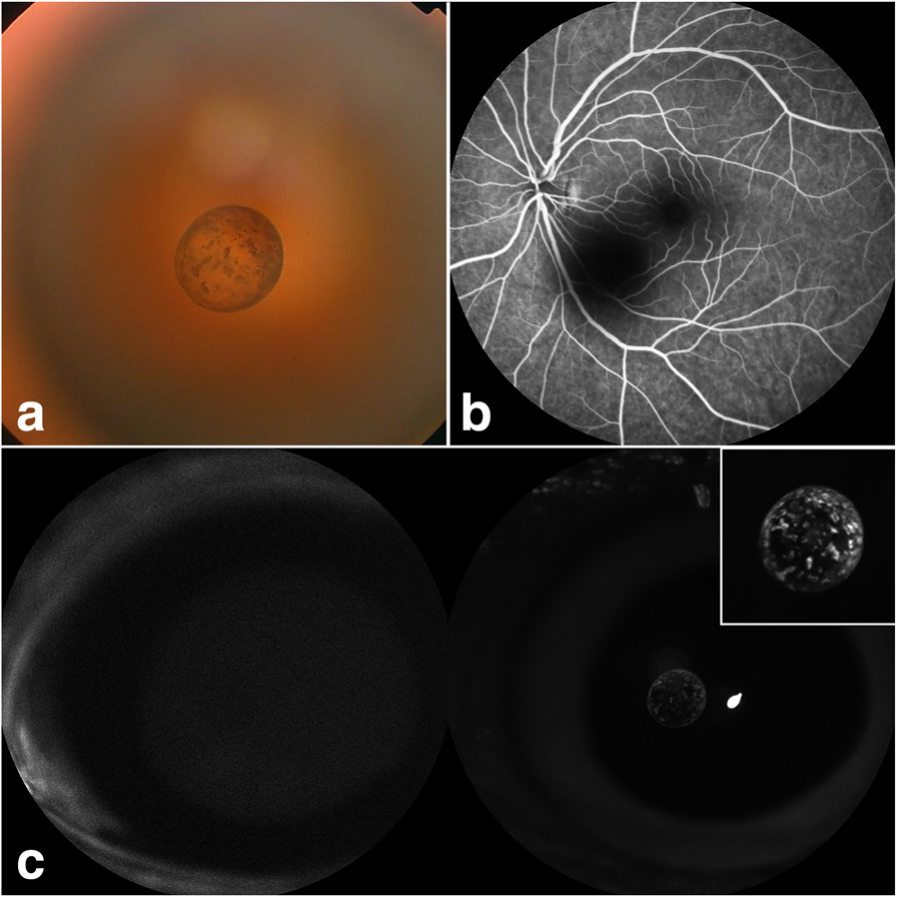
**a** Fundus photograph showing the pigmented vitreous cyst. **b** Fundus fluorescein angiography showing the cyst without connection to retinal and choroidal vessels. **c** The pigment clumps were absent of autofluorescence (left) but appeared hyperreflective in the corresponding infrared image (right)

The color of the pigment clumps on the vitreous cyst was yellowish-brown as revealed by anterior segment photography (Fig. 1a). The vitreous cyst was confirmed to be cystic without scolex and non-lobulated by AS-OCT (Visante™ OCT, Carl Zeiss) (Fig. 1b). No cyst or mass in the iris and ciliary body was detected by ultrasound biomicroscopy that was performed in twelve meridians (Fig.1c). No connection between the vitreous cyst and ocular structures was detected by B-scan ultrasound (Fig. 1d). Fundus examinations were performed to further testify the nature of the cyst (Fig. 2a-d). The cyst showed scattered and well demarcated pigmentation by fundus photograph (Fig. 2a). Fundus fluorescein angiography (Spectralis, Heidelberg Engineering) (Fig. 2b) ruled out the presence of persistent hyaloid artery. The pigment clumps in the cyst wall lacked autofluorescence by blue-light autofluorescence imaging (Fig. 2c, left) but appeared hyperreflective in the corresponding infrared image (Fig. 2c, right) (Spectralis, Heidelberg Engineering). Auxiliary tests were done to rule out other possible pathogenies. Results of the blood tests were normal. Serum tests for cysticercoids, sparganosis and toxoplasma were negative. Stool examination showed no parasite ova.

The above mentioned medical history, ophthalmic examinations, and auxiliary tests ruled out other possibilities such as parasitic infection and led to the diagnosis of an idiopathic pigmented vitreous cyst. The cyst has been followed up for one year without giving any interventions. At the latest follow-up, the cyst sank to the inferior part of the vitreous and the patient’s visual acuity remained stable with less frequent obscurations.

## Discussion

Vitreous cysts are generally classified into congenital and acquired ones. Non-pigmented congenital cyst may be remnants of the hyaloid vascular system such as Bergmeister’s papilla and Mittendorf’s dot [9], while pigmented congenital cyst may represent choristoma of the hyaloid vascular system [7, 10]. Acquired cysts are in conjunction with a variety of associations, such as retinitis pigmentosa, chorioretinal atrophy, and retinal detachment surgeries [1]. The acquired pigmented vitreous cysts have been reported to be associated with retinoschisis and uveal coloboma [11, 12]. In other cases, the etiology of the pigmented vitreous cysts could not be determined and such cysts were referred to as idiopathic [1, 6, 13–15]. A previous electron microscopic study on an idiopathic pigmented vitreous cyst has shown melanosomes in the pigmented cells, suggesting its pigment epithelial origin [6]. It was hard to tell whether the cyst originated from retinal or ciliary pigment epithelium, since that patient had a patch of lattice degeneration where the retinal pigment epithelial (RPE) cells could gain their access to the vitreous [6].

The vitreous cyst in our case was not secondary to ocular diseases or related to remnants of the hyaloid vascular system. Therefore, a diagnosis of idiopathic vitreous cyst was made. A new clinical feature of the pigmented cyst reported in this case was its absence of autofluorescence, indicating the lack of lipofuscin, a byproduct of the phagocytosis of shed photoreceptor outer segments [16]. Normally, the vitreous doesn’t have RPE cells, but only holds a few hyalocytes, astrocytes, and glial cells [17]. Given that no preexisting ocular diseases (neither inflammatory, degenerative, nor traumatic) were found and the retina was intact without any degeneration, it was highly unlikely that such RPE cells might migrate from the retina to the vitreous. Thus, the pigmented cells on the cyst wall were more likely to originate from the ciliary pigment epithelium. The cyst might be initially formed on the ciliary body and then dislodged into the vitreous, giving rise to the abrupt symptom of a huge floater. And its location in the anterior vitreous also strengthened our hypothesis.

Although laser and surgery are optional for symptomatic free-floating vitreous cysts, they were only preformed when visual disturbance have been lasted for several years [2, 6, 7]. From the follow-up visits of our case, it can be learned that the newly developed visual obscuration caused by a freely mobile cyst can be temporary and a period of observation may be recommended before resorting to any invasive methods.

## Conclusion

The pigmented vitreous cyst was idiopathic in this case. The intact retina, the absence of lipofuscin of the cyst and its location in the anterior vitreous led to the hypothesis that the cyst may originate from the ciliary pigment epithelium rather than the retinal pigment epithelium. AS-OCT can be useful in detecting pigmented vitreous cysts located at the anterior vitreous. For management of newly symptomatic free-floating vitreous cysts, a period of observation may be recommended before invasive intervention.

## Abbreviations

AS-OCT: Anterior segment optical coherence tomography
Nd:YAG: Neodymium:yttrium-aluminium-garnet
OD: Oculus Dexter
OS: Oculus Sinister
OU: oculus uterque
RPE: retinal pigment epithelial

## References

[1] Cruciani F, Santino G, Salandri AG (1999). Monolateral idiopathic cyst of the vitreous. Acta Ophthalmol Scand.

[2] Awan KJ (1985). Biomicroscopy and argon laser photocystotomy of free-floating vitreous cysts. Ophthalmology.

[3] Desai RU, Saffra NA (2010). Argon Laser Photocystotomy of a Vitreous Cyst. Ophthalmic Surg Lasers Imaging.

[4] Gupta R, Pannu BK, Bhargav S, Narang S, Sood S (2003). Nd:YAG laser photocystotomy of a free-floating pigmented anterior vitreous cyst. Ophthalmic Surg Lasers Imaging.

[5] Ruby AJ, Jampol LM (1990). Nd:YAG treatment of a posterior vitreous cyst. Am J Ophthalmol.

[6] Orellana J, O'Malley RE, McPherson AR, Font RL (1985). Pigmented free-floating vitreous cysts in two young adults. Electron microscopic observations. Ophthalmology.

[7] Nork TM, Millecchia LL (1998). Treatment and histopathology of a congenital vitreous cyst. Ophthalmology.

[8] Asiyo-Vogel MN (1996). el-Hifnawi el S, Laqua H. Ultrastructural features of a solitary vitreous cyst. Retina.

[9] Francois J (1950). Pre-papillary cyst developed from remnants of the hyaloid artery. Br J Ophthalmol.

[10] Jethani J, Maria N, Shetty S, Vijayalakshmi P (2007). Pigmented free-floating retrolental space cyst. J Cataract Refract Surg.

[11] Lavric A, Urbancic M (2013). Floating vitreous cyst: two clinical cases. Case Rep Ophthalmol.

[12] Tuncer S, Bayramoglu S (2011). Pigmented free-floating vitreous cyst in a patient with high myopia and uveal coloboma simulating choroidal melanoma. Ophthalmic Surg Lasers Imaging.

[13] Aydin E, Demir HD, Tasliyurt T (2009). Idiopathic pigmented free-floating posterior vitreous cyst. Int Ophthalmol.

[14] Gupta SR, Gupta N, Anand R, Dhawan S (2012). Idiopathic pigmented vitreous cyst. Arch Ophthalmol.

[15] Ludwig CA, Leng T (2016). Idiopathic pigmented vitreous cyst. Acta Ophthalmol.

[16] Fleckenstein M, Schmitz-Valckenberg S, Holz FG, Ryan SJ, Sadda S, Hinton D (2013). Autofluorescence Imaging. Retina.

[17] Coupland SE (2008). The pathologist's perspective on vitreous opacities. Eye (Lond).

